# Ontogeny of movement patterns in naïve grey seal pups inhabiting a complex continental shelf ecosystem

**DOI:** 10.1371/journal.pone.0290707

**Published:** 2023-09-27

**Authors:** Benia V. R. Nowak, W. Don Bowen, Cornelia E. den Heyer, Shelley L. C. Lang, Damian C. Lidgard

**Affiliations:** 1 Biology Department, Life Science Centre Dalhousie University, Halifax, Nova Scotia, Canada; 2 Population Ecology Division, Bedford Institute of Oceanography, Dartmouth, Nova Scotia, Canada; 3 Northwest Atlantic Fisheries Centre, St. John’s, Newfoundland, Canada; MARE – Marine and Environmental Sciences Centre, PORTUGAL

## Abstract

Most vertebrate offspring must transition from the relative security of parental care (nutrition and protection) to independent foraging. Offspring face many challenges during this critical period, particularly in species where parental care ends at weaning, such as the grey seal (*Halichoerus grypus*). We studied the development of movement behaviour in naïve grey seal pups from their first trips to sea to about five months of age. Twenty-five (12 males and 13 females) newly-weaned pups were fitted with satellite-linked GPS tags on Sable Island, Nova Scotia, Canada in January 2016. The influence of fixed effects (pup size, sex, week) and the random effect of pup identity on trip characteristics were examined. Movement behaviour was analyzed using a move persistence mixed-effects model. Habitat use was highly variable among individuals and covered much of the geographic distribution of the population. Unlike older juveniles, subadults, and adults in this population, most naïve pups used multiple haulout sites to begin and end trips. There was little evidence of area-restricted search behaviour during trips, suggesting that naïve pups were using an opportunistic foraging tactic that may result in more variable foraging success than that of older, experienced animals. Naïve pups made longer trips with longer haulout durations between them than observed for older greys seals. Males and females differed in some trip characteristics, but sex effects were small over the first few months of life. Offspring size at weaning was not a useful predictor of trip characteristics. Move persistence of grey seal pups was initially high and then decreased over time as individuals gained experience. Both intrinsic and extrinsic factors were influential on the movements of grey seal pups. Greater body length at weaning, longer duration spent on shore after weaning, shallower water column depth, and farther distance from shore were all associated with lower move persistence. Female grey seal pups had lower move persistence than males. Overall, the movements of naïve grey seal pups during the first few months of life were characterized by extensive exploration, but move persistence decreased over time suggesting they may be using an exploration-refinement foraging tactic.

## Introduction

The survival of offspring to reproductive age, and thus the fitness of both offspring and their parents, is dependent on the ability of offspring to learn to forage and avoid predation. In species that provide parental care, the survival of offspring at early life stages is largely reliant upon parental investment, which may include provisioning of food, supervision, teaching of learned behaviours, protection, or transportation [1]. However, offspring must eventually learn to forage independently. In some species, the transition from parental care to independent foraging occurs gradually (e.g., ursids, ungulates, primates; [2]) with some level of continued parental investment, while in others this transition occurs rapidly (e.g., small mammals, some ungulates, pinnipeds; [3]) and offspring must quickly learn to forage independently in order to survive. The duration of parental care may vary with both intrinsic (e.g., body size or energy stores) and extrinsic (e.g., resource availability and environmental conditions) factors, and thus the relative preparedness of their offspring for independent foraging may also vary.

To improve the chances of offspring survival, parents in capital breeding species transfer large quantities of stored energy (i.e., blubber or other fat deposits) to dependent offspring [4, 5]. Although this stored energy provides offspring with energetic reserves to support the transition to independent foraging, offspring nonetheless only have a limited amount of time to develop the skills needed to acquire food and avoid predation (e.g., [6, 7]). Size- and age-specific physiological constraints together with inexperience make the transition to independent foraging in juveniles a crucial period with important consequences for survival, dispersal, and recruitment to the breeding population [8]. As mortality is often high during this transition period [9–11], the development of behaviours promoting survival are critical and are likely to be under strong selection [12].

The use of telemetry devices provides an invaluable opportunity to study the development of behaviours *in situ*. These instruments have been particularly useful in the marine environment where observations have otherwise been limited to activities occurring at the surface [13]. Until recently, telemetry devices have often been too large to safely be attached to juveniles of most marine species resulting in fewer telemetry studies conducted on juveniles than older age classes (e.g., < 10% for seabirds and marine mammals [14]). Thus, substantial gaps in our understanding of the factors that drive the development of movement patterns and influence survival early in life still exist [15]. The miniaturization of electronic devices together with advancement of both telemetry technology (e.g., Fastloc GPS) and the statistical methods used to analyse these data (e.g., state-space models, hidden Markov models) have resulted in a growing number of studies that explore the ontogeny of movement patterns in marine species (e.g., [16–19]).

In pinnipeds, juvenile foraging effort is constrained by morphological and physiological limitations on dive depth and duration imposed by limits in the amount of oxygen that can be stored relative to metabolic demands [20]. As a result, juvenile and adult pinnipeds exhibit differences in dive depth and duration [21–24], forage in different areas [25, 26] and, thus, often have different diets [27, 28]. While studies have used pup movement data to address the ontogeny of diving from a physiological perspective [29], comparatively few have investigated the ontogeny of movement patterns and foraging behaviour [15].

Grey seals provide an interesting opportunity to study the ontogeny of foraging behaviour. At weaning, stored fat accounts for an average of 37.1% of the pup’s total body mass [30]. Thus, pups have considerable stored energy to support the transition to independent foraging. Yet, there is substantial variation in weaning mass [31] and length [32] among individuals, both of which influence survival to reproductive age and recruitment to the breeding colony [32]. Grey seal pups are weaned abruptly at approximately 17 days of age when their mothers return to sea to replenish depleted energy stores [30, 31]. Weaned pups remain on shore and undergo a post-weaning fast of up to several weeks before going to sea for the first time [33]. Thus, grey seal pups receive no parental guidance for what constitutes food, where to find it, or how to capture it. Although some behaviours must be under genetic control (namely food is only found at sea), other aspects of foraging behaviour would appear to rely on learning and experience.

Grey seals occur on both sides of the Atlantic and mainly inhabit continental shelves and inland seas [34–36]. Within these habitats, adults can routinely dive to the ocean floor [37, 38] and, as such, may be able to use topographical features to find food (as suggested be Wyles et al. [39]), aid in navigation, and to determine the offshore boundary of continental shelf habitats. There is evidence from the Northeast Atlantic (e.g., [17, 40, 41]) that grey seal pups rapidly develop foraging behaviours similar to those observed in adults over the first few months of life. However, differences in prey assemblages, predation pressure, and topographical characteristics in the Northwest Atlantic (i.e., deeper water column and greater distances between land masses) may influence the ontogeny of foraging behaviour.

The Scotian Shelf is a large, topographically complex marine ecosystem within the Northwest Atlantic which is comprised of a series of banks and basins [42]. These features influence the hydrographic properties of the region as cooler, fresher water from the Gulf of St. Lawrence becomes coastally-trapped and flows over the Scotian Shelf to form the top layer of this stratified shelf sea [43, 44]. The inflow of warmer, more saline waters from the shelf slope occurs through deeper channels but, due to density gradients, are largely unable to flow above the shallow banks [43, 45, 46]. These complex circulation patterns vary three-dimensionally and influence the distribution of prey species across the Scotian Shelf [47].

Sable Island is located on the outer edge of the Scotian Shelf and supports the largest breeding colony of grey seals in the world [48]. The number of pups born each year and total population size have continued to increase for over half a century [49, 50]. Associated with this increase in population size has been a substantial reduction in survival to recruitment (age four) [51]. Although sex differences in first year survival probability for animals born on Sable Island is not known, data from the Scotland population have indicated that first year survival was three times greater for females than males (53). Previous research on this population [26] as well as those in Wales and Scotland [17, 40] have demonstrated sex-specific differences in foraging behaviour, even prior to the onset of sexual size dimorphism. This research has also shown that foraging behaviour is influenced by oceanographic features such as water column depth and distance to shore, among others [26, 52, 53]. In this population, the foraging trips of older juvenile grey seals (five to twelve months) already closely resemble those of adults [26], yet how these characteristic patterns are developed is unknown.

The objective of this study was to explore the ontogeny of foraging behaviour in recently-weaned grey seal pups during their first months at sea. As changes in movement behaviour likely reflect learning (related to both foraging and predator avoidance) as individuals gain experience, we investigated how movement patterns changed over time and the influence of both intrinsic and extrinsic factors during this life stage. As male pups are heavier and longer than female pups [32, 54], we tested the effect of sex and body size on characteristics of trips to sea (i.e., trip frequency, trip distance, trip duration, and haulout duration) and how trip characteristics changed over time. As pups remain at the breeding colony post-weaning, they undergo both neurological and physiological changes during this period [33]. The post-weaning duration influences departure mass, resulting in a trade-off between physiological development and development of foraging skills. Variation in grey seal dive behaviour has been linked to post-weaning duration [7]. Here, we included post-weaning duration to account for these effects. Finally, we investigated the influence of intrinsic (i.e., sex, weaning body mass and length, and post-weaning duration) and extrinsic habitat characteristics (i.e., water column depth and distance to shore) known to influence the movement patterns of other age classes [26] on the foraging behaviour of naïve grey seal pups as well as its development over time.

## Methods

### Study site and data collection

**Fig 1:**
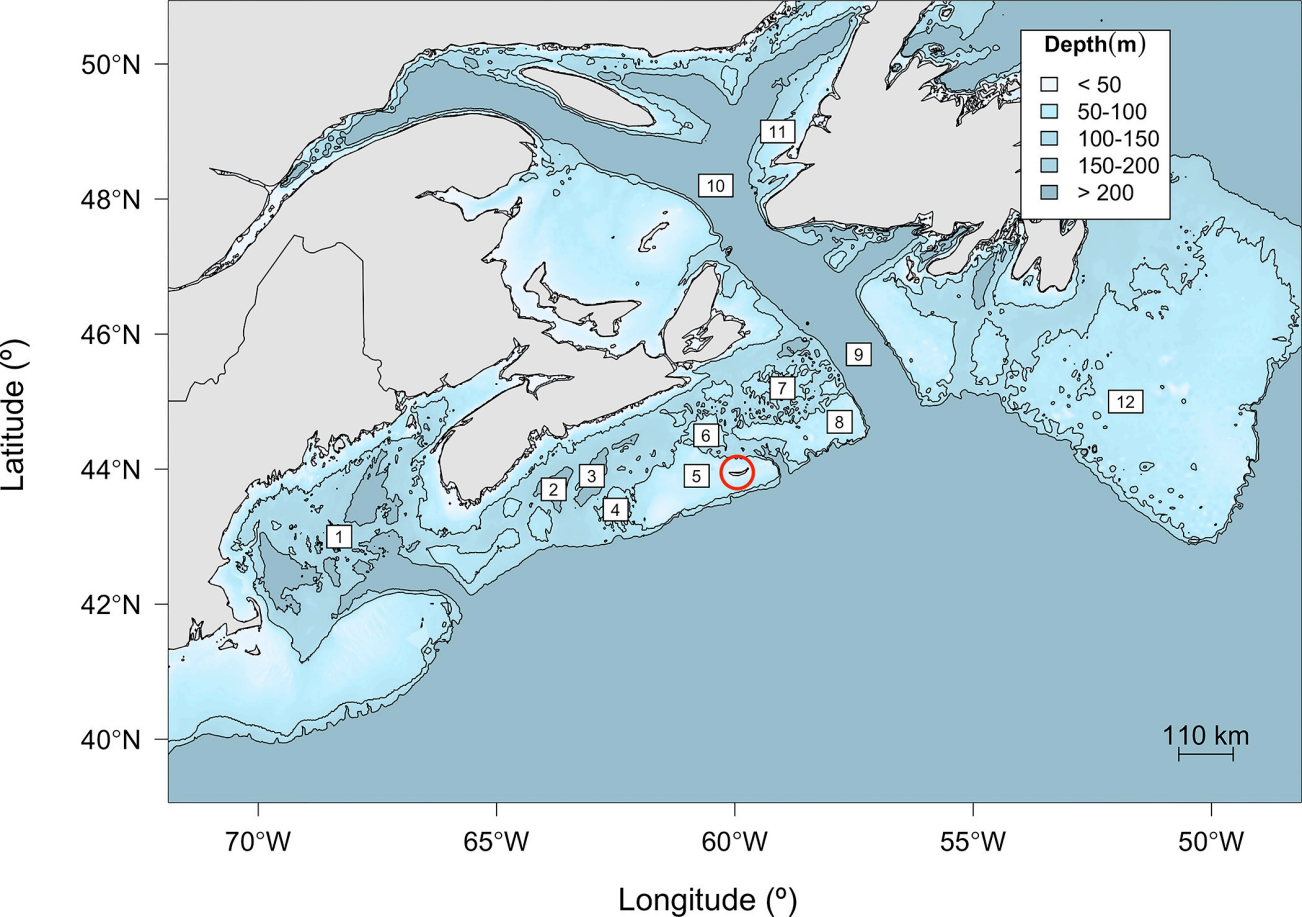
Bathymetry of the Scotian Shelf and surrounding regions with the 100 m and 200 m isobaths (black lines), Sable Island (red circle), and topographical features mentioned in text: (1) Gulf of Maine (2) LaHave Basin, (3) Emerald Basin, (4) Emerald Bank, (5) Sable Bank, (6) Middle Bank, (7) Misaine Bank, (8) Banquereau Bank, (9) the Laurentian Channel, (10) Gulf of St. Lawrence, (11) Western Shelf, and (12) Grand Banks. Bathymetric data acquired from the NOAA ETOPO1 database [59].

Our study was conducted on Sable Island (43.93° N, 59.91° W), a crescent-shaped sandbar 43 km in length and 1.5 km wide, located on the Scotian Shelf approximately 300 km southeast of Halifax, Nova Scotia, Canada (Fig 1). All sampling protocols were conducted in accordance with the requirements of the Canadian Council on Animal Care and were approved by Fisheries and Oceans Canada’s Maritime Region Animal Care Committee (protocol number 15–27) and Dalhousie University’s Committee on Laboratory Animals (protocol number 15–115). Work was undertaken under the Parks Canada Agency Research and Collection Permit SINP-2012-12974. Twenty-five (12 males and 13 females) recently-weaned grey seal pups were captured at the breeding colony in January 2016 using a small pole net. These pups were selected from a larger pool of identifiable individuals who had each been flipper tagged three or more days after birth. Female grey seals wean their pups by abruptly leaving the colony and returning to sea. To determine weaning date, marked adult females and their pups were sighted daily, but not disturbed. Body mass at weaning was then measured (to the nearest 0.5 kg) on the day following the female’s departure from the colony and the sex of the pup was recorded. For two pups weighed after the weaning date, body mass at weaning was estimated using a daily mass loss of 0.5 kg d^-1^ [33].

Following weaning, each of the 25 pups selected for the study were moved to a pen where they moulted their lanugo. Fully moulted pups were sedated with an intravenous injection of diazepam (5 mg/mL; 20 mg, Sandoz Canada Inc.) administered via the extradural vein. While sedated, standard dorsal body length was measured, sex confirmed, and a satellite-linked data transmitter (SPLASH 10-AF, www.wildlifecomputers.com) was glued to the dorsal pelage of the neck, following the methods of Breed et al. [34]. The instruments used in this study were repurposed from ones recovered during previous studies on this population, such that the battery life was reduced. The duration of penning varied among pups and depended on the percentage of lanugo that needed to be shed before the instrument could be applied; none were held for more than five days. Pups were not penned if they had completely moulted by the weaning date. Pups were released back into the colony near the pen site within an hour of sedation.

### Data processing

All data processing and statistical analyses were conducted using R 4.2.2 [55]. Satellite-linked tags were programmed to transmit Argos and Fastloc GPS locations to Service Argos. Fastloc locations were attempted after every 15 minutes when the animal was at the surface and were stored onboard tags until they were transmitted. Fastloc locations were suppressed during haulout periods when the wet/dry sensor was dry for 45 seconds of each minute for 20 consecutive minutes; tags exited the haulout state if the wet/dry sensor was wet for 45 seconds of one minute. Although both Argos and Fastloc locations were transmitted, we used Fastloc locations as they are more precise and do not include location error [56], there were a similar number of locations transmitted (25,475 Argos and 23,326 Fastloc locations; Table 1), and both would require regularization in time. Fastloc locations were filtered prior to analysis to remove inaccurate locations by removing locations which used fewer than five satellites [56, 57] and those which had a residual value greater than 30 [53, 56, 57]. A speed filter of 10 km h^-1^ was also applied to remove any erroneous locations when recorded swim speeds would not be possible [58]. A total of 1,926 locations were removed leaving 21,400 locations for further analysis.

**Table 1:** Summary of data collected for 23 of 25 naïve grey seal pups instrumented with SPLASH 10-AF satellite-linked transmitters (www.wildlifecomputers.com) on Sable Island during the 2016 breeding season. Tags deployed on two pups failed to transmit. Data includes sex (male = 1 and female = 2), body mass and length at weaning, post-weaning duration between weaning date and first day at sea, the total number of days of tracking between the date the tag was attached and the last day of transmitted data, number of Fastloc and Argos locations recorded, and number of complete trips. For two pups not weighed on the weaning date, body mass at weaning was estimated using a daily mass loss of 0.5 kg d^-1^ [33].

						Number		
Seal ID	Sex	Mass (kg)	Length (cm)	Post-Weaning Duration (d)	Deployment Duration (d)	Fastloc Locations	Argos Locations	Trips
13682	1	54.0	110	29	69	552	1001	4
13711	1	60.0	111	30	124	1601	1868	3
13783	1	50.5	107	24	38	324	545	2
13785	1	58.5	113	25	75	1347	1217	1
13804	1	54.0	110	19	84	1269	1342	2
13805	1	56.5	110	24	78	597	838	2
13827	1	61.5	117	27	111	1408	1560	4
13829	1	53.0	111	26	11	117	188	0
13831	1	62.0	118	20	52	547	753	1
13843	1	46.5	110	16	83	867	1103	0
13871	1	47.0	111	12	54	463	547	6
13882	2	41.5	108	20	53	647	901	0
13915	2	54.0	108	30	17	205	277	1
13916	2	57.0	112	17	174	3047	2675	9
13922	2	57.0	110	22	76	561	944	2
13939	2	64.0	114	7	46	357	594	2
13949	2	55.5	110	26	149	1743	2056	7
13969	2	56.0	115	23	118	1377	1476	3
13978	2	51.0	112	22	138	2667	2052	9
13985	2	62.0	110	22	122	922	1173	10
13987	2	55.0	116	13	40	217	361	0
13988	2	55.0	113	19	31	278	346	1
14587	2	NA	110	NA	160	2213	1928	11

### Trip characteristic analysis

Previous research on this population has demonstrated that nearly all movements of juvenile, subadult, and adult grey seals are organized into trips with clear behavioural switches including an outbound segment from Sable Island, one or more foraging patches (area-restricted search behaviour), and an inbound segment returning to Sable Island [26, 52]. Visual inspection of movements made by grey seal pups in this study showed marked differences in their overall structure, whereby individuals did not exhibit clear behavioural switches or segments and frequently hauled out at non-Sable Island locations. Filtered Fastloc locations were separated into individual trips using time periods when an animal was identified as hauled out. Haulouts were considered to be when any one of the following conditions applied: (i) a location was recorded as a haulout by the tag (ending when the tag exited the haulout state), (ii) the water column at a location was ≤ 5 m deep, or (iii) locations were within a 10 km radius surrounding Sable Island. Bathymetric data were acquired from the NOAA ETOPO1 database [59] using the R package *marmap* [60]. We used a conservative threshold of 5 m to account for any minor discrepancies in depth data used (e.g., due to haulouts on nearshore rocks, influence of tides, resolution of coastline) to ensure all haulouts were recorded. A trip was operationally defined as time at sea exceeding 24 hours [26]. A total of 20,022 locations were considered to be part of trips. Trips that did not end in a haulout (i.e., when the tag stopped transmitting mid-trip) were considered incomplete and could not be included in trip characteristic analyses (Table 1). For each complete trip, total duration (d), cumulative distance travelled between successive locations (km), and mean speed across tracks (km h^-1^) were calculated. Haulout duration (h) following each trip was also calculated (time between the first location classified as hauled out following a trip to the first location at-sea of the subsequent trip; S1 Table). Fastloc locations were then used to calculate the duration of the post-weaning period (the difference between the weaning date and first at-sea location of the first trip to sea). All locations were assigned a week at sea beginning with one on the first at-sea location of the first trip and the total number of weeks at sea was calculated based on the last at-sea location of each trip. The mean ± standard deviation was used to describe variation within our data.

To explore the influence of intrinsic factors on trip characteristics we conducted linear mixed-effects modelling with the R package *lme4* [61] using maximum likelihood estimation. One individual was omitted from analyses as biometric data were missing (i.e., weaning mass and post-weaning duration). Weaning mass and length were correlated (0.55, *p*-value < 0.01), but not to an extent that multicollinearity was a problem. We fitted linear models to assess the influence of sex, weaning body mass, and body length on trip frequency (i.e., number of trips made corrected for number of days), trip distance, trip duration, and haulout duration. The models for trip distance, trip duration, and haulout duration all included weeks at sea (at the end of each trip) and the interaction between sex and week as fixed effects. The model for haulout duration also included trip distance and trip duration as well as interaction terms with sex as fixed effects. Response variables were modelled assuming independent random normal errors (*εij* = *N*(0, *σ*^2^)). Pup identity was included as a random effect (ϒ*i* = *N*(0, *d*^2^)). The assumptions of a linear regression were tested using residual inspection and diagnostic tests. To meet assumptions, trip duration, trip distance, and haulout duration were ln-transformed. The best supported model was considered to be the model with the lowest second-order Akaike Information Criterion (AIC_c_) among models with all possible combinations of predictor variables. We also compared the log-Likelihood (LL) of the model with the lowest AIC_c_ to that of the model with the highest LL using likelihood ratio tests to confirm our selection.

### Move persistence analysis

We initially attempted to model the movements of naïve pups using the R package *swim* [62] which fit well to the tracks of adult grey seals in this population [53] and used the same process equation as Breed et al. [26, 52]. However, parameter estimates of this discrete formulation and visual inspection of model fits suggested that movement behaviour in these naïve pups was more continuous and, thus, discrete states could not be reliably estimated using the *swim* model.

Therefore, to explore the development of movement behaviour, we fitted the move persistence mixed-effects model (mpmm) implemented in the R package *mpmm* [63]. This model uses time-varying move persistence (γ_t_) estimated using autocorrelation in both speed and direction as a behavioral index that varies continuously between 0 (low move persistence, i.e., apparent area-restricted search) and 1 (high move persistence, i.e., apparent directed travelling). This continuous index provides a tool for describing behaviours, rather than discretizing them into distinct behavioural states [52]. High move persistence values (γ_t_ > 0.70) may be inferred as directed travel, whereas low move persistence values (γ_t_ < 0.30) may be indicative of area-restricted search [26, 64]. This model also allows for the evaluation of both group and between-individual variability in the relationships of move persistence with fixed effects through the inclusion of random effects. This model requires regularization of locations in time.

Prior to regularization, Fastloc locations were separated into shorter tracks to avoid prediction of move persistence when individuals were hauled out (see trip characteristic analysis section) or interpolation of locations over gaps in transmission > 48 h (e.g., due to satellite availability; n = 1) and assigned a track number. Tracks with fewer than 50 locations were not included [19, 65]. To determine the most suitable time step (time between regularized locations) for regularization of Fastloc locations within tracks, we assessed a series of possible time steps. To regularize locations in time, individual tracks were linearly interpolated at 3 h, 4 h, 6 h, and 8 h time steps. The null model (random intercept only) was then fitted for each time step using bounds-constrained quasi-Newton optimization (nlminb) with the Broyden-Fletcher-Goldfarb-Shanno algorithm and restricted maximum likelihood parameter estimates were used as initial values for maximum likelihood optimization to improve convergence; standard error estimates of γ_t_ were assessed and one-step-ahead residuals were calculated. The amount of time between observed Fastloc locations within tracks (i.e., data available for linear interpolation) was also calculated (third quartile = 2.27 h; 95% quantile = 7.22 h) to supplement comparison of these time steps. Although null models were run at 3 h and 4 h time steps and did not have convergence issues, these models were not useful as one-step ahead residuals indicated high levels of residual autocorrelation and the inclusion of predictors resulted in poor model fits (e.g., all γ_t_ ~ 1). The 3 h time step model also resulted in a much higher standard errors for move persistence estimates than all other models. The standard errors for move persistence estimates and one-step-ahead residuals suggested null models at both the 6 h and 8 h time steps fitted well and so 6 h time step was used for fitting move persistence models.

To assess the influence of habitat characteristics on the foraging behaviours of grey seal pups water column depth and distance to shore (the shortest great-circle distance to the 0 m isobath) were calculated for each interpolated location. Locations were then assigned a week number based on the number of weeks since the first day at sea. This coarser temporal scale (rather than days at sea) was used given the time step of interpolated locations and allowed for interpretation at time scales when greater change may be observed. In addition to habitat characteristics, the influence of intrinsic factors was also considered and one individual was omitted from analyses as biometric data were missing. A total of seven potential explanatory variables were included in move persistence modelling: sex, body mass (kg) and length (cm) at weaning, post-weaning duration (d), water column depth (m), distance from shore (km), and week number. Interactions between explanatory variables were not tested as the inclusion of interaction terms is not currently available within the mpmm framework. Given the short duration of deployments for several individuals included in this study, we aimed to summarise the influence of intrinsic and extrinsic factors over the initial trips to sea. All models included random intercepts for individual pups, but random slopes were not included due to model fit and convergence issues. We fitted all possible combinations of fixed and random effects using bounds-constrained quasi-Newton optimization (nlminb) with the BFGS algorithm. To improve convergence, restricted maximum likelihood parameter estimates were used as initial values for maximum likelihood optimization allowing for model comparison. The lowest AIC_c_ and highest LL were used to determine the best-supported model to estimate move persistence [63, 65].

One-step-ahead residuals were calculated and used to check model assumptions and assess goodness-of-fit for the best-supported model (S1–S3 Figs). The move persistence model employs an unstructured covariance matrix rather than a first-order autoregressive covariance structure [63] which was suitable as there was little residual autocorrelation observed in our data (S1 Fig). Independence was assessed using Variance Inflation Factors which were < 2 for all predictors, providing no evidence of multicollinearity [66]. To meet model assumptions, water column depth was ln-transformed prior to model fitting (S3 Fig). Interpolated locations at or above 0 m depth (maximum 1.9 m above surface) were assigned a depth of 1 m to reflect proximity to shore and permit the use of ln-transformation. To assess performance of the best-supported model, leave-one-out cross validation was used where each individual included in the model was iteratively left out, the model was run at each iteration, and the coefficient estimates relative to the full model including all individuals were compared [65]. This included the quantile range (5% and 95%) for the model including all individuals and the estimated trend for the percentage of cross-validation models that fell within the 95% confidence intervals of the model including all individuals.

## Results

Twenty-five newly-weaned grey seal pups were instrumented with satellite-linked transmitters over a 10-day period near the end of the 2016 breeding season (Table 1). As expected, males tended to be both heavier (55.7 ± 4.74 *vs*. 54.5 ± 6.31 kg) and longer (112.0 ± 3.38 *vs*. 111.2 ± 2.37 cm) than females on average, but these differences were small (t-test, *p*-value = 0.61 and *p*-value = 0.51, respectively) in this sample. Time spent on shore post-weaning prior to the first trip ranged from 3 to 26 d, with males spending about 5 d longer on land than females (23.9 ± 4.25 d *vs*. 19.1± 6.56 d; t-test, *p*-value = 0.057). There was no correlation between weaning mass and the duration of the post-weaning fast (0.087, *p*-value = 0.70) consistent with previous studies of grey seals [7, 33].

**Fig 2:**
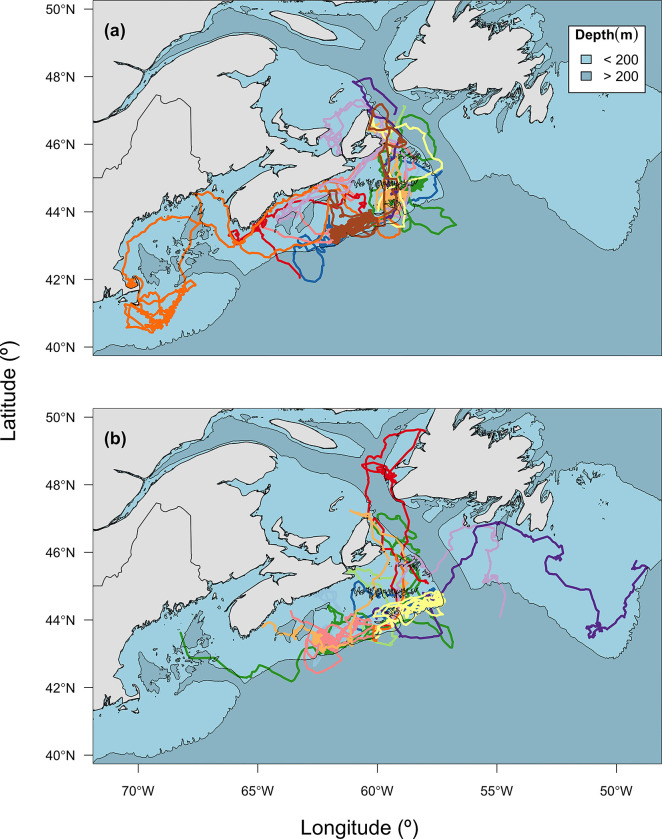
Fastloc GPS tracks of naïve grey seal pups instrumented with SPLASH 10-AF satellite-linked transmitters (www.wildlifecomputers.com) on Sable Island during the 2016 breeding season separated by sex where (a) female tracks (n = 12) and (b) male (n = 11). Individuals pup tracks are represented by unique colours with the 200 m isobath included (black lines) to highlight the edge of the continental shelf. Bathymetric data were acquired from the NOAA ETOPO1 database (NOAA 2009).

None of the pups left the island immediately after being released, suggesting that penning was unlikely to have influenced the timing of their initial departure to sea. Previous research has demonstrated that grey seal pups on Sable Island move extensively throughout the breeding colony during the post-weaning period [33]. Therefore, the location of release should not have influenced their subsequent behaviour. Of the 23 pups that left Sable Island with functioning transmitters, 17 did so from the south beach even though they were released near the center of the island. Four of the six that left from the north beach immediately swam to the south side of the island. Thus, pups showed a marked preference for waters to the south of Sable Island during their first trip to sea (Fig 2).

The satellite transmitters used in our study had all been used previously and, therefore, we expected the periods of transmission generally not to exceed three to five months. Nevertheless, 74% of the tracks covered most of the first two months following the departure from the natal colony, a period during which we expected the most rapid learning might occur [17], and 35% of the tracks covered the first three to six months of life. Instrumented naïve pups transmitted data for an average of 82.7 ± 46.38 days following their first trip to sea, resulting in an average of 930 ± 776 filtered Fastloc locations. Pups moved mostly within the offshore portion of the eastern Scotian Shelf. The inshore portion of the shelf also was used but somewhat less intensively and more so by female than male pups (Fig 2). Although most pups limited their movements to the Scotian Shelf, two pups moved into the Gulf of Maine to the south of the Scotian Shelf and five moved north across the deep Laurentian Channel into the Gulf of St. Lawrence or onto St. Pierre Bank or the Grand Banks off southern Newfoundland (Fig 2). At some point in their tracks, 22 out of 23 pups were located over waters deeper than 200 m (i.e., near the shelf slope or over shelf basins; [42]), for a mean of 18.6 ± 15.96 locations per individual or about 6.3 days. Movements over the deep ocean (i.e., beyond 1000 m) lasted only an average of 3.0 ± 1.71 d.

### Trip characteristics

The structure of naïve grey seal pup trips was highly variable compared to those of juveniles, subadults, and adults (Table 2). Of 23 pups that transmitted locations at sea, 19 individuals (9 males, 10 females) made complete trips (S1 Table). The four pups that did not return to land after going to sea for the first time spent from 11 to 83 days at sea (S1 Table). Of the 80 complete trips made by the 19 pups, about half (10 pups) made a total of 38 return trips to Sable Island, 13 pups made 16 trips between Sable Island and a non-Sable Island haulout, and 8 pups made 26 trips between two non-Sable Island haulout locations. Fifteen of the 19 pups with three or more trips made multiple trip types.

**Table 2:** Trip characteristics comparing < 5-month-old pups (this study) to all other age classes [26, 37] including trip duration (d), surface speed (km h^-1^), distance to shore (km), water depth (m), and haulout duration (h). For consistency with previous research, mean ± standard errors [26] are presented for all response variables and if needed, standard deviations were converted to standard errors using reported sample sizes [37]. Surface speed presented for < 5-month pups includes all at-sea locations but for other age classes includes only travel segments of trips.

Response variable	Adult males	Adult females	Subadults	5 to 12-month pups	< 5-month pups
Trip duration (d)	7.4 ± 1.0	6.5 ± 0.6	5.1 ± 1.0	8.6 ± 1.3	15.9 ± 0.9
Surface speed (km h^-1^)	1.5 ± 0.2	1.4 ± 0.1	1.7 ± 0.3	1.6 ± 0.2	1.7 ± 0.03
Distance to shore (km)	45.1 ± 4.3	39.6 ± 5.3	29.9 ± 7.4	53.8 ± 8.2	91.1 ± 3.2
Water depth (m)	52.4 ± 4.0	38.9 ± 12.9	41.5 ± 19.1	39.9 ±13.7	143.7 ± 15.7
Haulout duration (h)	20.8 ± 1.1	12.5 ± 0.7			33.6 ± 1.5

The number of trips made varied greatly among individuals (S1 Table) but was largely dependent on the lifespan of the tag (r = 0.79, *p*-value < 0.001). Of the pups that made trips, males made fewer trips on average (2.3 ± 0.6) than females (4.6 ± 1.2). This remained the case when corrected for the number of days transmitting (0.03 trips/d males vs 0.10 females). Trips were highly variable in both duration and distance lasting on average 15.9 ± 16.6 d (CV = 105%) and covering a total distance of 594.5 ± 594.2 km (CV = 100%). Overall, the average horizontal movement speed during trips was 1.7 ± 0.5 km h^-1^. The duration of haulouts between trips was also quite variable, lasting on average 33.6 ± 29.0 h (CV = 86%; S1 Table).

The trip frequency model including sex and mass as predictors (marginal R^2^ = 0.48; Table 3) provided the lowest AIC_c_ and while it had the second-highest LL, the LL was not significantly different compared to the full model which had the highest LL (*Χ*^*2*^ = 0.21, *p* = 0.64). Female grey seal pups had significantly higher trip frequencies than males and those with lower weaning masses had higher trip frequencies (Table 3). The best-supported models for both trip distance (Table 4) and trip duration (Table 5) included both sex and weeks at sea. These models provided the lowest AIC_c_ scores but did not have the highest LLs. However, the LL for both the trip distance (*Χ*^*2*^ = 2.84, *p* = 0.42) and trip duration (*Χ*^*2*^ = 2.49, *p* = 0.48) models were not significantly different compared to the full models including interaction terms which had the highest LLs. Some iterations of the trip duration model showed evidence of singularity which suggested the influence of the random effect was negligible. The best supported models suggested females had lower trip distances and durations than males and that the higher the number of weeks at sea, the farther the trip distances and longer the trip durations. However, both the trip distance (conditional R^2^ = 0.25) and trip duration (conditional R^2^ = 0.27) models fitted poorly and explained only a small amount of the variance in the data. Thus, none of the fixed effects measured in this study were good predictors of either estimated trip duration or distance travelled from the trip origin over the first four months of life. The best-supported haulout duration model included only mass as a predictor, however it explained only a small amount of the variance in the data (conditional R^2^ = 0.39; Table 6). This model resulted in the lowest AIC_c_ and while it did not have the highest LL, the LL was not significantly different compared to the model with the highest LL (*Χ*^*2*^ = 2.85, *p* = 0.94). Nevertheless, the fixed effects were not particularly useful predictors of haulout duration.

**Table 3:** Model selection of trip frequency (number of trips/days at sea) for 18 naïve grey seal pups with pup sex, pup body mass and length at weaning as fixed effects and pup identity as a random effect. The number of degrees of freedom (df), log-Likelihood (LL), second-order Akaike Information Criterion (AIC_c_), difference in AIC_c_ from that of the best-supported model (ΔAIC_c_), and Akaike weight (*w*_*i*_) are presented. The best-supported model is indicated in bold. Coefficient estimates, standard errors, and *p*-values of predictor variables are shown for the best-supported model.

Model structure	df	LL	AIC_c_	ΔAIC_c_	*w*_*i*_
**~ sex + mass**	**4**	**46.24**	**-81.40**	**0.00**	**0.64**
~ sex	3	43.53	-79.35	2.06	0.23
~ sex + mass + length	5	46.35	-77.69	3.71	0.10
~ 1	2	39.84	-74.88	6.53	0.02
**Coefficient**	**Estimate**		**Std. Error**		***p*-value**
Intercept	0.179		0.064		0.013
sex (female)	0.0278		0.0097		0.012
mass	-0.00256		0.0011		0.037

**Table 4:** Model rankings by the change in the Akaike information criterion (ΔAIC_c_) and log likelihood for the linear mixed-effects model for ln-transformed trip distance (km) fit to trip data from 18 naïve grey seal pups. The number of degrees of freedom (df), log-Likelihood (LL), second-order Akaike Information Criterion (AIC_c_), difference in AIC_c_ from that of the best-supported model (ΔAIC_c_), and Akaike weight (*w*_*i*_) are presented. The best-supported model is indicated in bold. Coefficient estimates, standard errors, and t-values of predictor variables are shown for the best-supported model.

Model structure	df	LL	AIC_c_	ΔAIC_c_	*w*_*i*_
**~ sex + week + (1 | id)**	**5**	**-99.38**	**209.71**	**0.00**	**0.42**
~ week + (1 | id)	4	-101.04	210.71	1.01	0.26
~ sex + length + week + (1 | id)	6	-98.88	211.11	1.40	0.21
~ sex + mass + length + week + (1 | id)	7	-98.87	213.58	3.87	0.06
~ sex + mass + length + week + sex*week + (1 | id)	8	-97.96	214.32	4.61	0.04
~ (1 | id)	3	-106.09	218.54	8.84	0.01
**Coefficient**	**Estimate**		**Std. Error**		**t-value**
Intercept	5.65		0.27		21.16
sex (female)	-0.57		0.29		-1.95
week	0.081		0.021		3.80

**Table 5:** Model rankings by the change in the Akaike information criterion (ΔAIC_c_) and log likelihood for the linear mixed-effects model for ln-transformed trip duration (d) fit to trip data from 18 naïve grey seal pups. The number of degrees of freedom (df), log-Likelihood (LL), second-order Akaike Information Criterion (AIC_c_), difference in AIC_c_ from that of the best-supported model (ΔAIC_c_), and Akaike weight (*w*_*i*_) are presented. The best-supported model is indicated in bold. Coefficient estimates, standard errors, and t-values of predictor variables are shown for the best-supported model.

Model structure	df	LL	AIC_c_	ΔAIC_c_	*w*_*i*_
**~ sex + week + (1 | id)**	**5**	**-96.47**	**203.89**	**0.00**	**0.55**
~ sex + mass + week + (1 | id)	6	-96.25	205.85	1.97	0.21
~ week + (1 | id)	4	-99.08	206.78	2.89	0.13
~ sex + mass + length + week + (1 | id)	7	-96.17	208.17	4.28	0.06
~ sex + mass + length + week + sex*week + (1 | id)	8	-95.22	208.85	4.96	0.05
~ (1 | id)	3	-106.86	220.09	16.21	0.00
**Coefficient**	**Estimate**		**Std. Error**		**t-value**
Intercept	5.06		0.25		20.64
sex (female)	-0.60		0.26		-2.33
week	0.10		0.020		4.80

**Table 6:** Model rankings by the change in the Akaike information criterion (ΔAIC_c_) and log likelihood for the linear mixed-effects model for ln-transformed haulout duration (h) fit to trip data from 18 naïve grey seal pups. The number of degrees of freedom (df), log-Likelihood (LL), second-order Akaike Information Criterion (AIC_c_), difference in AIC_c_ from that of the best-supported model (ΔAIC_c_), and Akaike weight (*w*_*i*_) are presented. The best-supported model is indicated in bold. Coefficient estimates, standard errors, and t-values of predictor variables are shown for the best-supported model.

Model structure	df	LL	AIC_c_	ΔAIC_c_	*w*_*i*_
**~ mass + (1 | id)**	**4**	**-99.00**	**206.66**	**0.00**	**0.55**
~ mass + week + (1 | id)	5	-98.86	208.72	2.07	0.19
~ (1 | id)	3	-101.41	209.21	2.55	0.15
~ mass + duration + week + (1 | id)	6	-98.59	210.61	3.95	0.08
~ mass + length + duration + week + (1 | id)	7	-98.53	212.99	6.33	0.02
~ sex + mass + length + duration + week + (1 | id)	8	-98.50	215.52	8.86	0.01
~ sex + mass + length + duration + distance + week + (1 | id)	9	-98.49	218.20	11.54	0.00
~ sex + mass + length + duration + distance + week + sex*duration + (1 | id)	10	-97.57	220.84	14.18	0.00
~ sex + mass + length + duration + distance + week + sex*duration + sex*distance + (1 | id)	11	-97.65	222.19	15.53	0.00
~ sex + mass + length + duration + distance + week + sex*duration + sex*distance + sex*week (1 | id)	12	-98.42	225.04	18.38	0.00
**Coefficient**	**Estimate**		**Std. Error**		**t-value**
Intercept	8.67		2.46		3.52
mass	-0.10		0.044		-2.36

### Move persistence

The most parsimonious model predicting move persistence was the full model including sex, body mass and length, post-weaning duration, week number, distance to shore, and water column depth as fixed effects (Table 7). Leave-one-out cross validation indicated that the best supported model performed well across all iterations (Table 7). Overall, estimates of move persistence were high but variable (0.69 ± 0.14), suggesting that movements were largely exploratory relative to those of adults. Nearly half of locations (2873 out of 5826 locations) did not exhibit move persistence values typical of behavioural states (0.30 ≤ γ_t_ ≤ 0.70). Move persistence values indicated that directed travel (i.e., γ_t_ > 0.70) may have occurred in all 22 pups and corresponded to 2918 locations. Only 35 locations among 8 pups were characteristic of typical area-restricted search behaviour (i.e., γ_t_ < 0.30).

**Table 7:** Model selection for the move persistence mixed effects model fitted to 22 naïve grey seal pup tracks interpolated at a six-hour time step and estimate move persistence (γ_t_). Fixed effects: week = week of tracking, body mass and length at weaning, postwean = time between weaning and first trip to sea, distshore = distance nearest 0 m isobath, and depth = ln-transformed water column depth. Pup identity is included as a random intercept. The number of degrees of freedom (df), log-Likelihood (LL), second-order Akaike Information Criterion (AIC_c_), difference in AIC_c_ from that of the best-supported model (ΔAIC_c_), and Akaike weight (*w*_*i*_) are presented. The best-supported model is indicated in bold. Coefficient estimates, standard errors, and *p*-values of predictor variables are shown for the best-supported move persistence mixed-effects model. Coefficients with significant *p-*values are indicated in bold. The quantile range (5% and 95%) of parameter estimates from leave-one-out cross validation of the most parsimonious model are presented with the estimated trend (Est Trend) which represents the percentage of cross validation models where the estimated coefficients fall within the 95% confidence intervals of the parameter estimates from the model including all individuals.

Model structure		df	LL	AICc	ΔAIC_c_	*w*_*i*_
**~ sex + mass + length + postwean + week + distshore + depth + (1 | id)**		**13**	**17473.49**	**-34920.91**	**0.00**	**0.96**
~ sex + length + postwean + week + distshore + depth + (1 | id)		12	17469.18	-34914.32	6.60	0.04
~ sex + length + postwean + distshore + depth + (1 | id)		11	17460.32	-34898.59	22.32	0.00
~ length + postwean + distshore + depth + (1 | id)		10	17453.32	-34886.60	34.31	0.00
~ length + distshore + depth + (1 | id)		9	17451.14	-34884.26	36.65	0.00
~ distshore + depth + (1 | id)		8	17449.32	-34882.61	38.30	0.00
~ depth + (1 | id)		7	17415.84	-34817.66	103.25	0.00
~ (1 | id)		6	17376.96	-34741.90	179.01	0.00
**~ sex + mass + length + postwean + week + distshore + depth + (1 | id)**			**Cross validation**			
**Coefficient**	**Estimate**	**Std Error**	***p*-value**	**5%**	**95%**	**Est Trend (%)**
Intercept	11.747	3.72	**< 0.01**	4.46	19.03	100
sex (female)	-0.452	0.18	**< 0.05**	-0.80	-0.10	100
mass	0.0151	0.018	0.39	-0.019	0.050	100
length	-0.105	0.036	**< 0.01**	-0.18	-0.035	100
postwean	-0.0507	0.017	**< 0.01**	-0.083	-0.018	100
week	-0.0355	0.0072	**< 0.0001**	-0.050	-0.021	95
distshore	-0.00525	0.00065	**<0.0001**	-0.0065	-0.0040	90
depth	0.502	0.041	**<0.0001**	0.42	0.58	95

**Fig 3:**
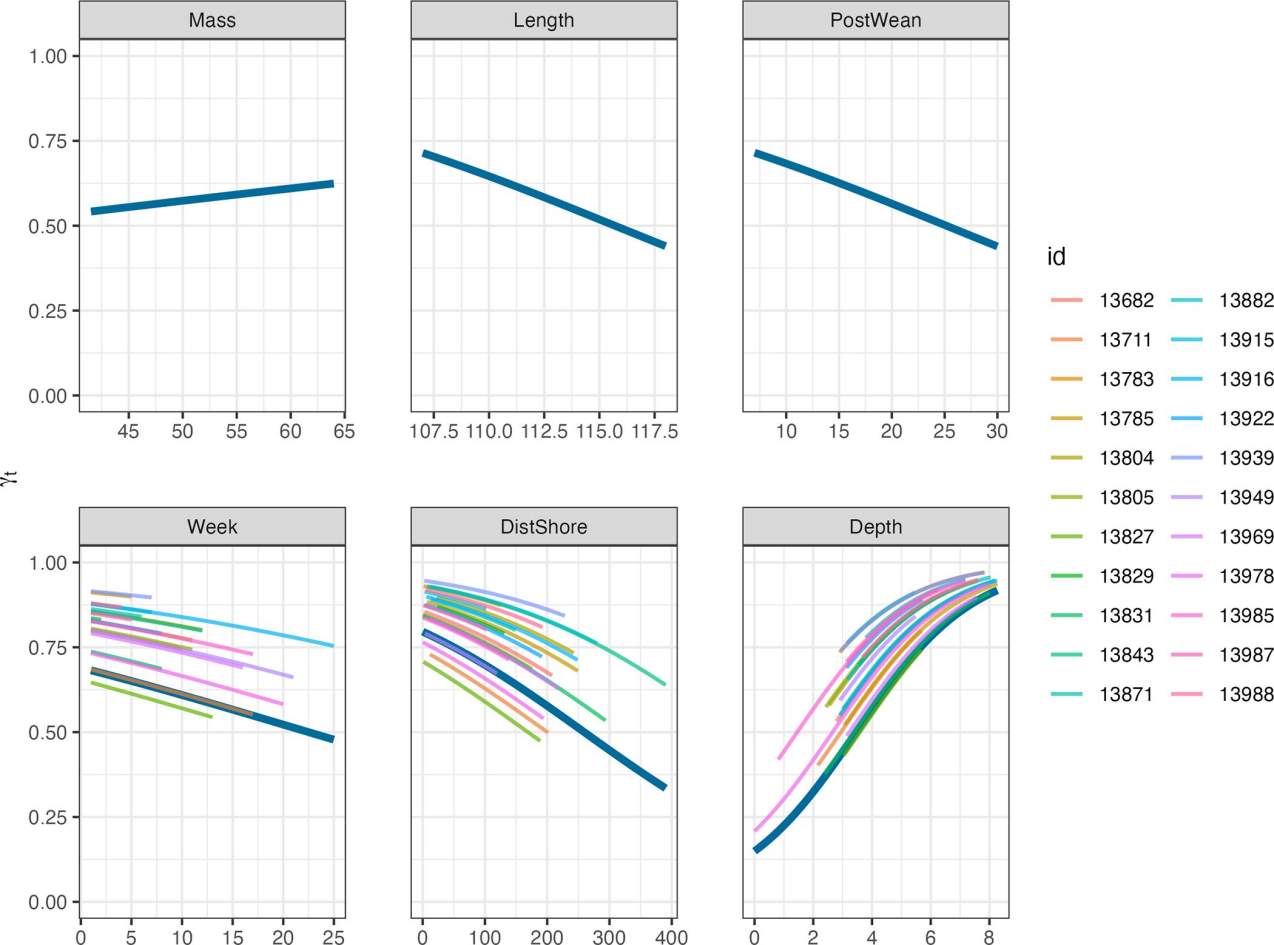
Move persistence estimates (γ_t_) from the best-supported move persistence mixed-effects model fitted to Fastloc GPS location data from 22 naïve grey seal pups interpolated at a six-hour time step. Plots display individual (coloured lines) random effects and group (blue line) fixed effects responses for continuous predictors and include weaning body **mass** (kg) and **length** (cm), the **post**-**wean**ing duration (d), **week** number, the **dist**ance to nearest **shore** (km), and ln-transformed water column **depth** (m); the effect of sex is not shown.

The three most influential variables (based on *p-*values) predicting move persistence were week, distance to shore, and water column depth (Table 7). With each passing week, grey seal pups tended to exhibit lower move persistence values, suggesting a change in behaviour to include more tortuous movement (Fig 3). As water column depth decreased (i.e., movement over shallower banks), individuals were more likely to exhibit lower persistence values (Fig 3). As grey seal pups moved farther away from shore, they exhibited lower move persistence values. Sex was an important predictor of move persistence and female grey seal pups demonstrated lower move persistence than males. Body length at weaning was also a significant predictor of move persistence and suggested that longer grey seal pups had lower move persistence values. Grey seal pups who had longer post-weaning durations (amount of time between the weaning date and first day at sea) tended to have lower move persistence values during their first few months at sea. Body mass at weaning was retained in the model however it was not a useful predictor of move persistence in naïve pups.

## Discussion

The analysis of foraging trip characteristics and move persistence indicate that the movement behaviour of recently-weaned grey seal pups differs from older grey seals in this population but develops rapidly during their first few months at sea to resemble that of adults. While the frequency with which trips were made was influenced by sex and weaning mass, trip characteristics were highly variable among individuals. Despite some support for the influence of sex and the number of weeks at sea on trip distance and duration, and the influence of weaning mass on haulout duration between trips, fixed effects were not good predictors of trip characteristics. There was little evidence of area-restricted search behaviour by these naïve pups, which is observed in older juveniles (age 5 to 12 months), subadults, and adults [26, 52, 53]. During the first few months at sea, move persistence estimates were high, suggesting largely exploratory behaviour. However, there was clear evidence of a decrease in move persistence over the first three to four months of life, suggesting a somewhat rapid change in behaviour as pups gained experience. Extrinsic factors including water column depth and distance to shore were the best predictors of the decrease in move persistence over time. Intrinsic factors including sex, post-weaning duration, and body length at weaning were also useful predictors for the move persistence model. Female grey seal pups demonstrated lower move persistence than males during their first few months at sea, consistent with sex-specific differences in foraging ontogeny observed in other grey seal populations [17, 40]. Grey seal pups with longer post-weaning durations tended to exhibit lower move persistence. This may be owed to the more advanced physiological development and dive capabilities these individuals may have relative to pups with shorter post-weaning durations. It is possible that this could also be due to increased pressure to acquire food following a longer fast prior to departure for sea. Body mass at weaning was included as a predictor in the best-supported model, however the effect was not significant. Although males are both heavier and longer than females at weaning, the difference is only about 5% [67]. Longer individuals were more likely to have lower move persistence which might indicate an early foraging advantage which may contribute to the positive effect of body length on survival to recruitment [32]. Longer pups may be able to dive deeper and longer [68, 69] and thus may have improved foraging success or ability to avoid predators, however these hypotheses remain to be tested.

### Development of foraging behaviour in pinnipeds

The rapid development of foraging skills is critical to survival as newly-weaned capital breeding pinnipeds have limited time to learn how to capture prey before their blubber reserves are depleted [7, 70, 71]. Variable first-year survival impacts recruitment into the breeding population and future reproductive success in several mammal species, including seals (e.g., [32, 72, 73]). Survival to recruitment (age 4) during the period of our study was less than 30% [51, 74]. Assuming that, like other large mammals, much of the subadult mortality in grey seals occurs in the first year of life [75], there is likely strong selection on the rapid acquisition of foraging skills.

Evidence of a rapid development in foraging ability seems widespread among pinniped species and comes from changes in both diving ability and movement behaviour. Within the first month or so at sea, pups of several species exhibit rapid increases in dive depth and duration and in the overall amount of time spent diving [7, 16, 17, 19, 69, 76, 77]. These are in part due to physiological development along with changes in body condition that affect buoyancy [78, 79]. Given that prey species are typically found at depth, these changes in behaviour probably reflect increasing foraging ability.

### Development of trip characteristics in Sable Island grey seals

The characteristics of foraging trips appear to change less quickly and are more variable than those related to diving behaviour observed in other pinniped species [24]. This may be related to the fact that foraging is constrained to a greater extent by physiological limits during dives than ranging behaviour and thus should be under greater selective pressure. Nevertheless, the initial direction of migration appears to be established early during the first foraging trip in male and female northern elephant seals (*Mirounga angustirostris*; [80]) and harp seals (*Pagophilus groenlandicus*; [19]), although in the case of harp seals the onset of migration was delayed compared to that of adults. The initial foraging trip of newly-weaned southern elephant seals (*Mirounga leonina*) also appears to be established early as it exhibits elements of adult trips, including a strong bias in the direction of travel [70]. The grey seal pups in this study also showed a strong preference for an initial direction of trip departure from the south side of the island. We speculate pups may use the southward shelf-break jet or the more gradual increase in water column depth present on the southern side of Sable Island to orient out to sea. The implication is that grey seal pups may be able to use environmental cues as has been reported in harbour seals [81] to orient themselves in space. However, this speculation remains to be investigated.

We found no evidence that changes in trip duration or distance were dependent upon intrinsic factors measured (e.g., body mass and length) aside from sex. In contrast to grey seal pups studied at Scottish and Welsh colonies [17], the effect of sex was only weakly supported given the trip characteristic models fitted poorly, those that excluded sex fitted nearly as well, and the interaction between sex and week was not retained in the final model. While grey seal pups in this study showed some evidence of changes in trip structuring with increasing experience (i.e., successive weeks at sea), the models explained only a small amount of the variation in the data such that it is not expected these characteristics change consistently over time. We speculate that to some degree these differences may be dependent on what is encountered during exploration (i.e., conspecific, predators, and prey) and how foraging success improves over time, but it could also reflect the longer period of tracking for the Scottish and Welsh seals (averaging up to 148 and 166 d, respectively). Carter et al. [17] found that evidence for a sex effect was variable depending on the metric considered in two regions around the UK. For example, the same level of sexual segregation in depth, proportion of bottom time, and trip duration that was observed in the Celtic and Irish Seas was not evident in the North Sea. The bathymetry and types of grey seal habitat differ between these regions, where the North Sea is less variable than the Celtic and Irish Seas. While the Scotian Shelf is highly variable, bathymetric features are often still deeper than naïve grey seal pups may be able to dive (e.g., shallow banks deeper than 50 m; Fig 1). These regional differences in habitat complexity may be influential on the sex-specific differences observed in pups.

### Development of move persistence in Sable Island grey seals

**Fig 4:**
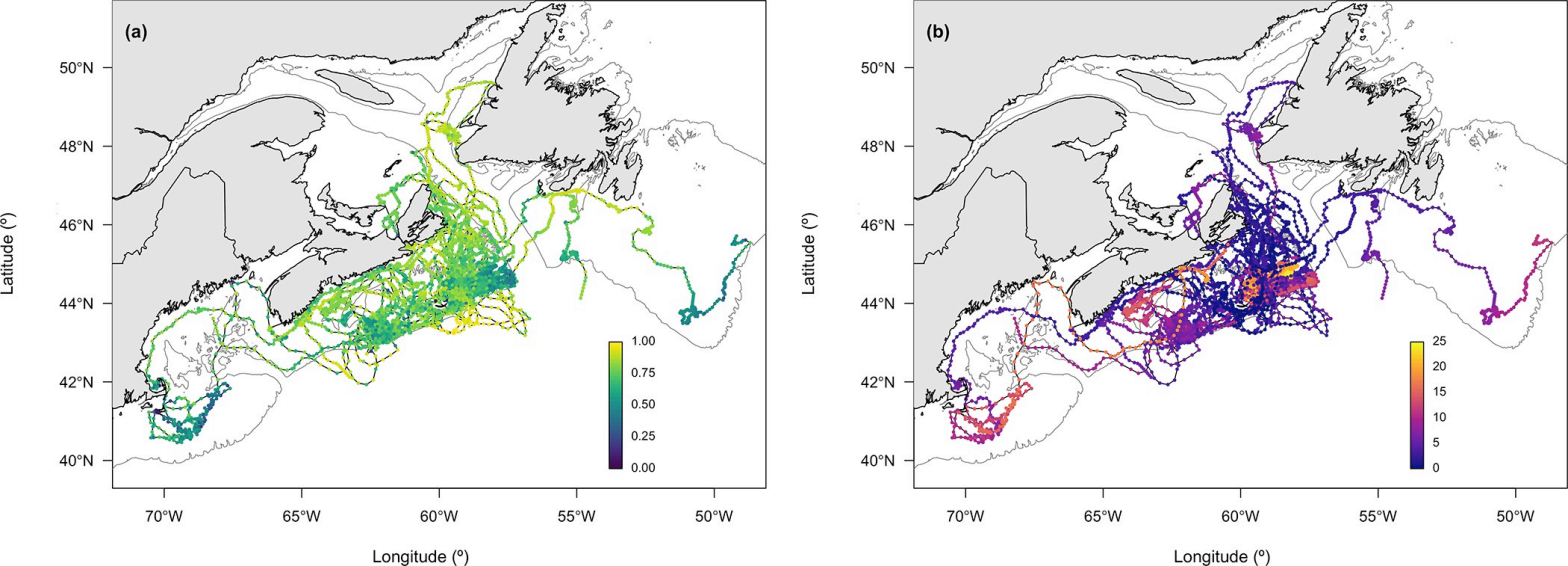
Fastloc GPS tracks from 22 naïve grey seal pups instrumented with SPLASH 10-AF satellite-linked transmitters (www.wildlifecomputers.com) on Sable Island during the 2016 breeding season showing locations interpolated at a six-hour time step coloured as a gradient for (a) move persistence (γ_t_) estimated using the best-supported move persistence mixed-effects model and (b) weeks at sea.

Move persistence of naïve grey seal pups during their first week at sea was high and variable among all individuals (Fig 3). Although there were only a small number of locations with move persistence estimates typical of area-restricted search [52], there was evidence of shifting between high and moderate move persistence along tracks suggesting that movements were more exploratory in nature than are commonly observed in adults. Nevertheless, our finding that move persistence decreased on average over the first few months at sea suggests that naïve grey seal pups gain experience rapidly and change their behaviour accordingly. With each passing week, naïve pups tended to exhibit lower move persistence values, demonstrating a change in behaviour to include more tortuous movement (Fig 3). It is possible that pups with longer transmission times were those that were more successful, and thus move persistence decreased on average because unsuccessful pups were no longer part of the data set. However, there was evidence of lower move persistence for a number of individuals occurring over shallow banks (Emerald Bank, Sable Bank, and Banquereau Bank; Fig 1) commonly used for foraging by adults from this population (Fig 4A; [53]). This is supported by both greater distance from shore and shallower water column corresponding to lower move persistence, as the foraging behaviour of adults in this population is largely concentrated over shallow offshore banks [53]. The use of these topographical features were also largely associated with greater time at sea (Fig 4B), indicating that through experience, individuals may have learned prey could be found in these areas, resulting in lower move persistence. Similar findings have been reported for naïve grey seal pups in both the Irish and Celtic Seas and the North Sea [40]. These results for grey seals along with those from both seabird (e.g., Cory’s shearwaters, *Calonectris borealis*; [82]) and other pinniped species (e.g., northern fur seals, *Callorhinus ursinus*; [24]) suggest that the exploration-refinement hypothesis may describe the development of foraging behavior. This hypothesis predicts that foraging behaviour is learned during individual exploratory behaviours early in life, which then become canalized with age and experience [83].

Another indicator of differences in behaviour between age class was the time step used to regularize location data. When we initially attempted to fit the null model to grey seal pups locations regularized at a 3 h time step, inspection of the one-step ahead residuals indicated high residual autocorrelation. It is possible that given the overall high move persistence estimates observed at the 6 h time step, exploratory search behaviours characterized by moderate move persistence could not be discriminated from travel at lower time steps. In contrast, adult grey seal locations regularized at a 3 h time step modelled using a hidden Markov model (HMM) with the same process equation as the move persistence model showed that locations could be classified into discrete behaviours and trips had well-defined outbound, apparent foraging, and inbound segments. This time step was selected based on known characteristics of foraging trips [53, 62]. While these differences may in part be due to the resolution of the location data used to fit models, move persistence estimates from the best-supported model fitted at a 6 h time step were variable between high and low move persistence which was not observed in juveniles, subadults, or adults [26], such that there are likely real differences in the characteristics of movement patterns between these age classes. Notably, studies on grey seal pups inhabiting other regions (i.e., the North Sea and Wales) have been able to resolve behavioural states using time steps of only one to two hours [40, 41]. Again, this difference could be related to the quality of location data in each of these studies or modelling approach used but could also reflect real differences in how naïve animals explore their environments.

### Comparison to other grey seal populations

Variation in foraging behaviour among individuals and populations is generally thought to be mediated by factors such as food availability, risk of predation, the physical structure of the environment in diverse taxa [84–87]. Therefore, a secondary objective of our study was to compare our results with the behaviour of grey seal pups in other grey seal populations to see if differences in habitat characteristics might influence the development of movement behaviour. In addition to the Sable Island breeding colony on the Scotian shelf (this study), the movement of naïve grey seal pups has been studied from breeding colonies in the northeastern United States [88], those adjacent to the Irish and Celtic Seas, and the northern North Sea [17, 40]. Slightly older pups (6–8 weeks of age) were tracked in the southern North Sea [41]. As different analysis methods and metrics of behaviour and study objectives were used across these studies (e.g., Murray et al. [88] focused on the spatial overlap between pup movements and fisheries and did not provide estimates of trip characteristics), it is difficult to draw firm conclusions, but several generalizations do seem to emerge. First, in each of these areas, pups ranged widely, exhibiting what appeared to be exploratory behaviour, but also exhibited considerable variability in movement among individuals with some remaining more local than others. Second, although the movements of naïve pups are wide-ranging, they appeared to sense the edge of the continental shelf beyond which there is no prospect of successfully capturing prey. Although the depth and duration of dives of grey seal pups improves rapidly [7], dives greater than about 50 m are uncommon and short [17]. Thus, once off shallow offshore banks, such as Sable Island, pups would be unable to dive to the seafloor either to forage or to gain cues for navigation, although occasionally deeper dives may be possible. In situations where naïve pups left the Scotian Shelf and travelled to locations where the depth exceeded 1000 m this was typically for periods of only few days before they invariably returned to the shelf and usually occurred during a pups’ first and second trips to sea. Similarly, few inexperienced grey seal pups studied in the other populations ventured into waters deeper than 100 m [17, 41, 88]. In addition to water depth, cues such as water temperature and salinity and characteristics of current may be important but remain to be investigated. Third, strong regional differences are evident in the trip duration and distance travelled. Of the populations studied, pups in the Celtic and Irish Seas had the shortest trips at about 2.5 days compared to trips of about 9–10 days for pups in the North Sea [17, 41] and on the Scotian Shelf (this study). Although distance travelled during trips also varies by geography, it is not simply a function of trip duration as pups from the southern North Sea and those in the Celtic and Irish Seas travelled similar distance despite having trip durations that were quite different. The mechanism responsible for these differences remains to be determined. We speculate that both the spatial extent of potential habitat, the distribution of food, and the potential for encountering other haulouts must play important roles.

### Comparison to other age classes

Although the longitudinal study of individuals may be the most direct way to gain insight into the ontogeny of foraging behaviour, comparison of the behaviour of young individuals with that of older conspecifics from the same population can also be valuable (e.g., [18, 26, 89]). Breed et al. [26, 52] modelled the behaviour of juveniles, subadults, and adults from the Sable Island population using similar methods to those used in this study. This allowed us to examine the ontogeny of movement behaviour by comparing characteristics of initial trips to those taken by more experienced, older grey seals (Table 2). This comparison showed that naïve pups tended to have longer trips than juveniles, subadults, and adults suggesting they may have had greater difficulty in finding food than older seals either due to lesser foraging ability or from the effects of intraspecific competition [90]. There is evidence that poorer foraging proficiency of young individuals results in more time spent foraging in European shags (*Phalacrocorax aristotelis*; [11]). Poor foraging proficiency of young may be related to reduced ability to capture and handle prey as found in oystercatchers (*Haematopus ostralegus*; [91]), but this remains to be investigated. Grey seal pups may also be foraging more opportunistically than older age classes, spending more time acquiring information about their environment and developing knowledge on suitable foraging habitat. The greatest differences in behaviour of naïve pups and older grey seals were associated with the habitat characteristics encountered, including distance to shore and water column depth (Table 2). Naïve pups travelled about two to three times further from shore than older animals and ventured over waters beyond the shelf break. We speculate that these differences in behaviour reflect the greater difficulty naïve pups are expected to have with finding and capturing prey. Similar differences have been reported in diverse taxa suggesting, as might be expected, that the lack of experience plays a large role in determining behaviour [89]. The longer haulout time between trips presumably reflects a greater need for pups to recover after having travelled longer at sea (Table 2). As observed in juvenile southern elephant seals, haulout duration may also reflect differences in body condition [78] which could not be assessed in the current study.

Bottom topography and the presence of conspecifics might also be used by pups to guide the development of their foraging behaviour. Phocid seals tend to dive continuously while travelling or foraging [37, 92]. Grey seals mainly inhabit continental shelves and inland seas [34–36]. Within these habitats, adults can routinely dive to the ocean floor [37, 38] and, as such, may be able to use bathymetric features to find food, aid in navigation, and to determine the offshore boundary of continental shelf habitats [39]. Apart from any genetic predisposition, naïve pups have little to guide them on where to locate food and, thus, we should expect their initial movements at sea to be exploratory. It is certainly possible that they may obtain some useful information on the location of prey from older conspecifics. However, following older conspecifics may not be profitable because pups are unlikely to have the necessary skills to capture the prey eaten by more experienced animals. Support for this comes from evidence of spatial segregation of pups and adults [26] suggesting that pups may be out-competed by older animals. Although the diet of naïve pups is not known, juveniles consume a diet comprising of greater species diversity than older animals presumably reflecting their more limited foraging ability [28]. As noted by Carter et al. [17], observations of foraging behaviour from animal-borne video or indirect methods such as accelerometry and stomach temperature telemetry will be needed to better understand the influence of environmental differences on the ontogeny of foraging behaviour.

## Supporting information

S1 Fig Auto-correlation function (ACF) plots of one-step-ahead residuals for longitude and latitude from the best-supported move persistence mixed-effects model. (PDF)

S2 Fig Histogram of one-step-ahead-residuals for longitude and latitude from the best-supported move persistence mixed-effects model. (PDF)

S3 Fig Plots of the one-step-ahead residuals for longitude and latitude from the best-supported move persistence mixed-effects model and the covariates week (top), distance to shore (middle), and ln-transformed depth (bottom). There are no obvious patterns between the residuals and covariates other than some evidence for unequal variance, which suggests that the linear assumption is adequate.
(PDF)

S1 Table Type and characteristics of individual trips performed by naïve grey seal pups (n = 19) instrumented with SPLASH 10-AF satellite-linked transmitters (www.wildlifecomptuers.com) on Sable Island in 2016. Trips were assigned to one of three types: (A) return trip to Sable Island (natal colony), (B) trip between Sable Island and a non-Sable Island haulout location, or (C) trip between two non-Sable Island haulout locations. Trip characteristics included total trip surface distance (km), trip duration (d), average surface speed (km h^-1^), and haulout duration (h). Not applicable (NA) values for haulout duration indicate that no subsequent trips were performed following the haulout.
(DOCX)
