# Supplementary figures and images for: Ontogeny of movement patterns in naïve grey seal pups inhabiting a complex continental shelf ecosystem
Source: PLoS One. 2023 Sep 27;18(9):e0290707. doi: 10.1371/journal.pone.0290707 (PMC10529606; doi:10.1371/journal.pone.0290707)

# Longitude

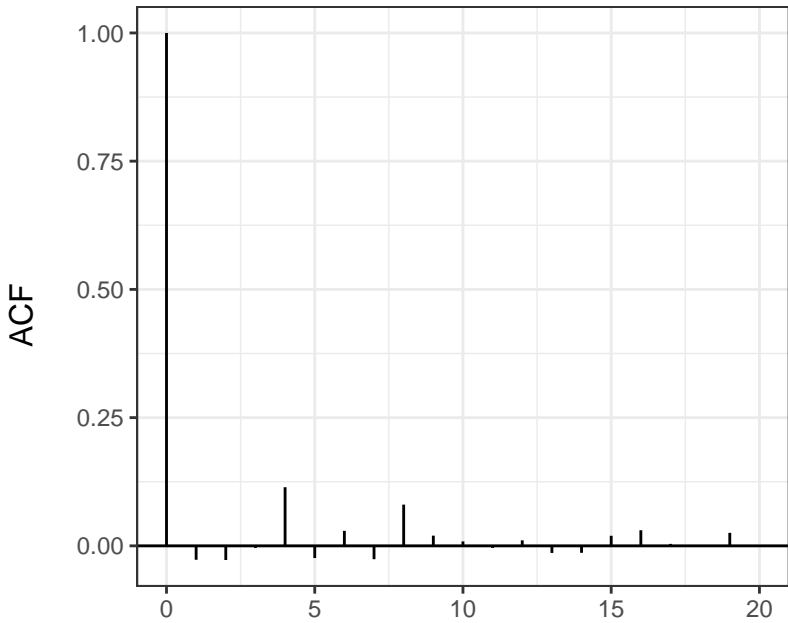

# Latitude

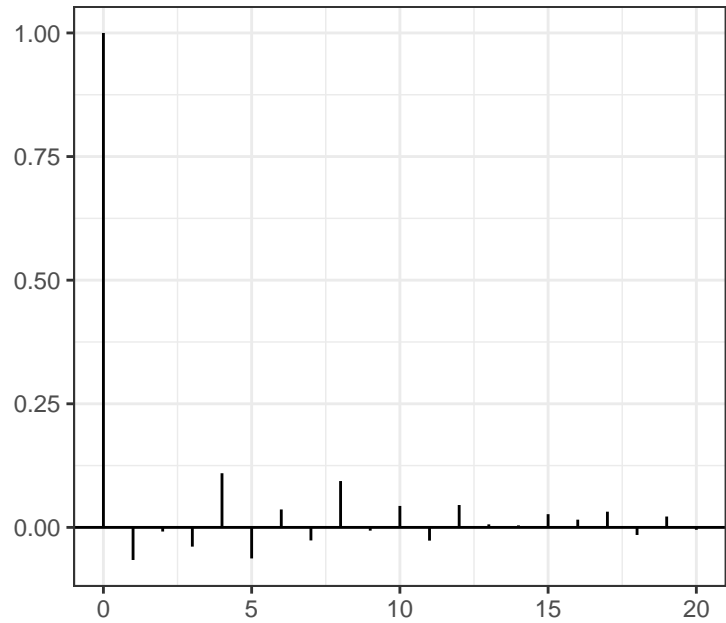

Supplement: S1 Fig — (PDF) [file pone.0290707.s001.pdf]

# Longitude

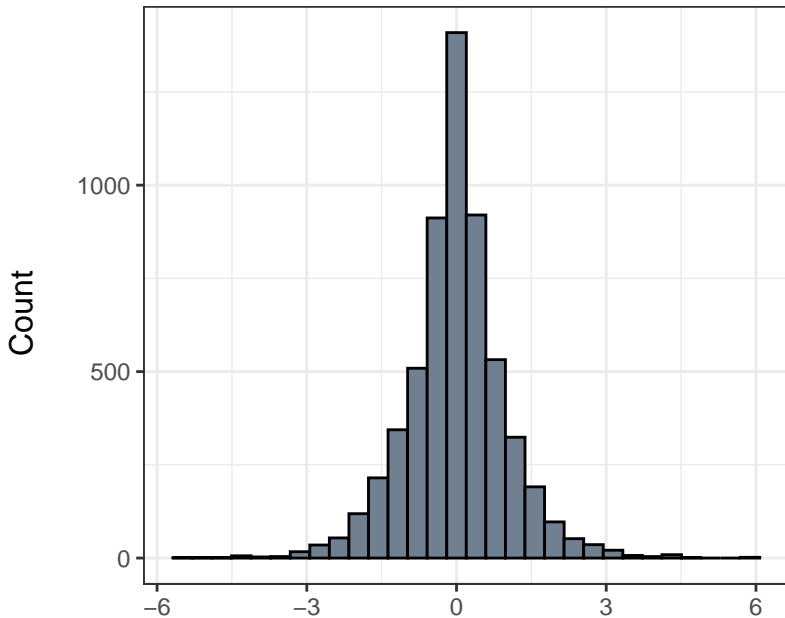

# Latitude

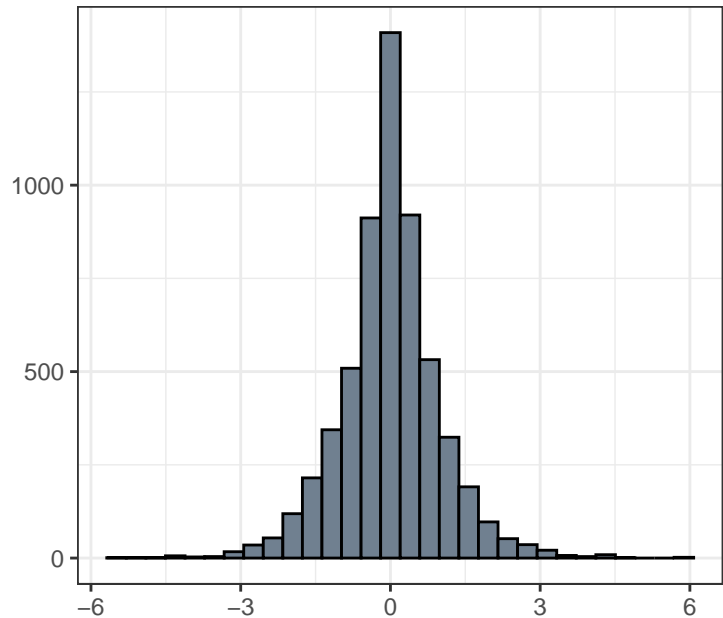

Residual

Supplement: S2 Fig — (PDF) [file pone.0290707.s002.pdf]

Longitude

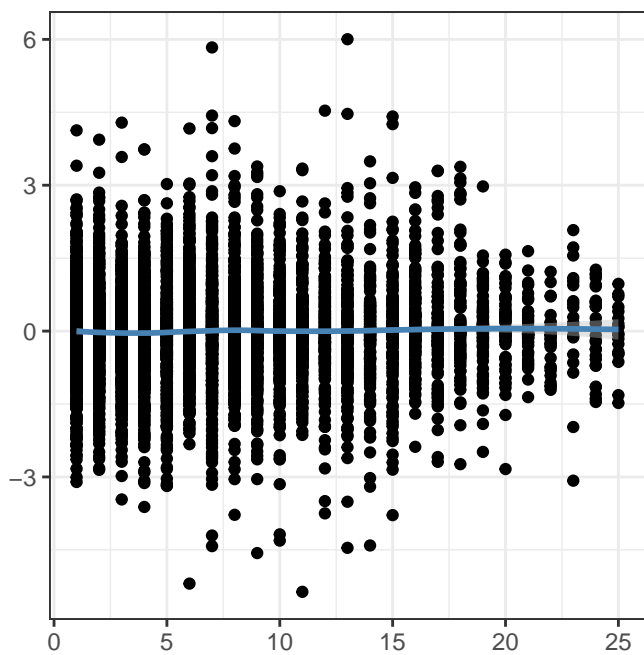

Latitude

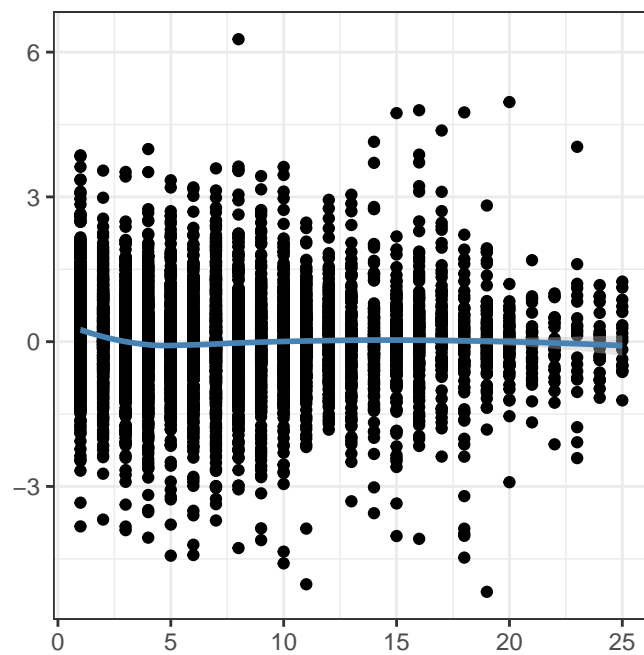

Residual

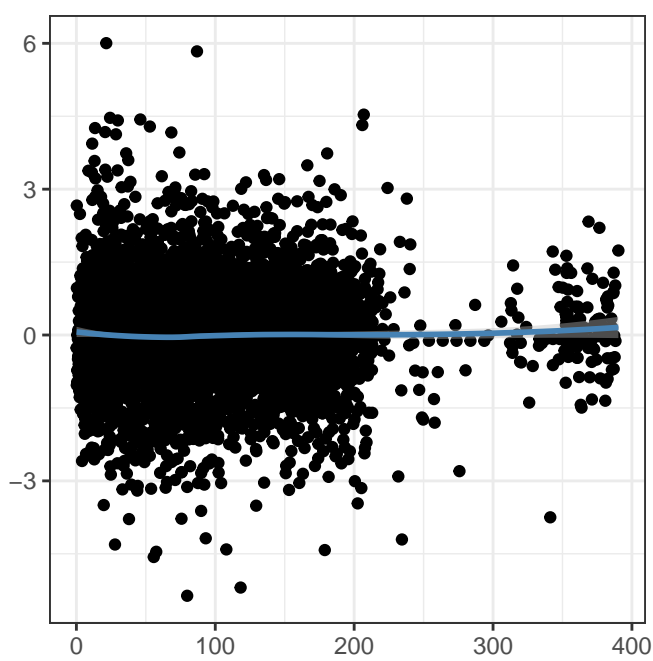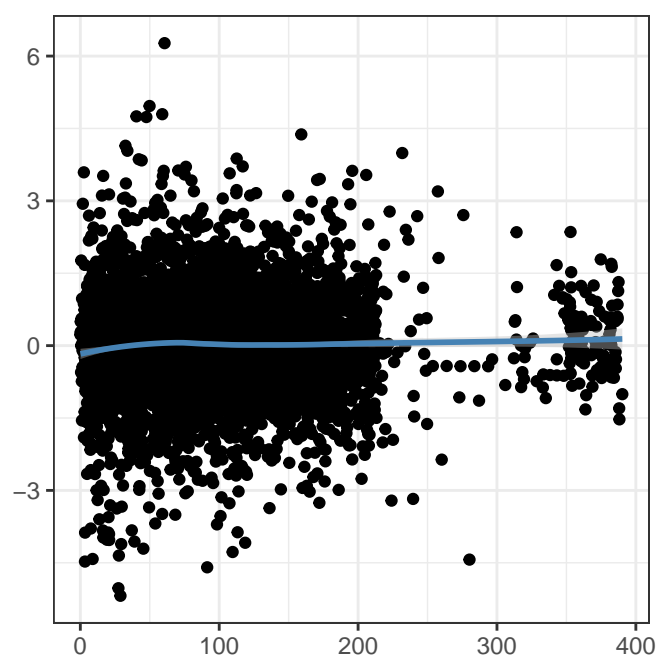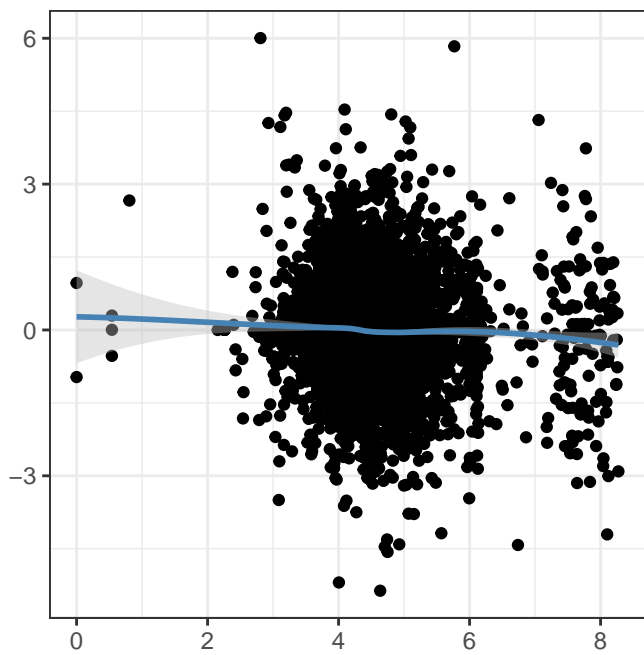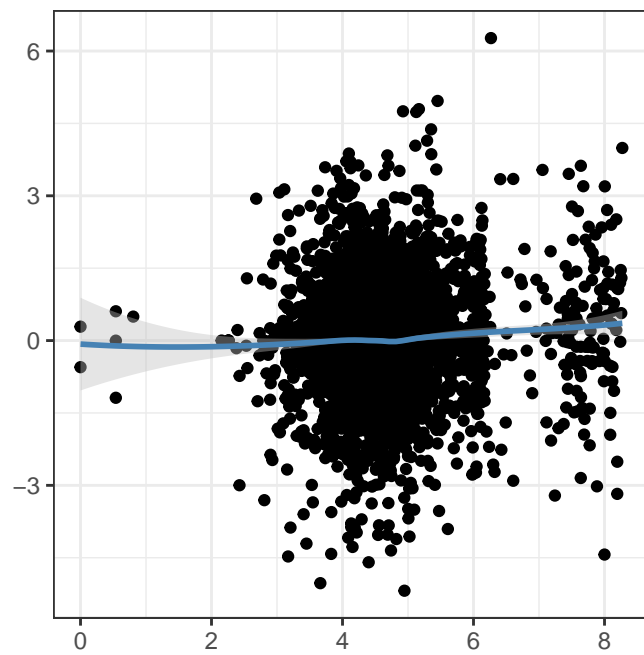

Value

Supplement: S3 Fig — Plots of the one-step-ahead residuals for longitude and latitude from the best-supported move persistence mixed-effects model and the covariates week (top), distance to shore (middle), and ln-transformed depth (bottom). There are no obvious patterns between the residuals and covariates other than some evidence for unequal variance, which suggests that the linear assumption is adequate. (PDF) [file pone.0290707.s003.pdf]
