# Supplementary material for: Ontogeny of movement patterns in naïve grey seal pups inhabiting a complex continental shelf ecosystem
Source: PLoS One. 2023 Sep 27;18(9):e0290707. doi: 10.1371/journal.pone.0290707 (PMC10529606; doi:10.1371/journal.pone.0290707)
Supplement: S1 Table — Trips were assigned to one of three types: (A) return trip to Sable Island (natal colony), (B) trip between Sable Island and a non-Sable Island haulout location, or (C) trip between two non-Sable Island haulout locations. Trip characteristics included total trip surface distance (km), trip duration (d), average surface speed (km h-1), and haulout duration (h). Not applicable (NA) values for haulout duration indicate that no subsequent trips were performed following the haulout. (DOCX) [file pone.0290707.s004.docx]

**S1 Table** Type and characteristics of individual trips performed by naïve grey seal pups (n = 19) instrumented with SPLASH 10-AF satellite-linked transmitters ([www.wildlifecomptuers.com](http://www.wildlifecomptuers.com)) on Sable Island in 2016. Trips were assigned to one of three types: (A) return trip to Sable Island (natal colony), (B) trip between Sable Island and a non-Sable Island haulout location, or (C) trip between two non-Sable Island haulout locations. Trip characteristics included total trip surface distance (km), trip duration (d), average surface speed (km h^-1^), and haulout duration (h). Not applicable (NA) values for haulout duration indicate that no subsequent trips were performed following the haulout.

| **Seal ID** | **Trip Number** | **Trip Type** | **Trip Distance (km)** | **Trip Duration (d)** | **Average Speed (km h^-1^)** | **Haulout Duration (h)** |
| --- | --- | --- | --- | --- | --- | --- |
| 13682 | 1 | B | 369.84 | 13.21 | 1.17 | 9.05 |
| 13682 | 2 | C | 64.30 | 2.39 | 1.12 | 2.55 |
| 13682 | 3 | C | 145.64 | 2.66 | 2.28 | 30.00 |
| 13682 | 4 | C | 43.26 | 1.44 | 1.25 | 13.15 |
| 13711 | 1 | A | 2565.46 | 76.12 | 1.40 | 29.20 |
| 13711 | 2 | A | 421.90 | 11.12 | 1.58 | 46.30 |
| 13711 | 3 | A | 387.61 | 14.06 | 1.15 | 21.82 |
| 13783 | 1 | B | 506.84 | 17.17 | 1.23 | 18.05 |
| 13783 | 2 | B | 570.93 | 12.55 | 1.90 | 65.46 |
| 13785 | 1 | A | 2435.88 | 42.14 | 2.41 | 12.39 |
| 13804 | 1 | A | 2492.29 | 69.31 | 1.50 | 105.30 |
| 13804 | 2 | B | 447.20 | 9.36 | 1.99 | NA |
| 13805 | 1 | B | 417.84 | 11.51 | 1.51 | 81.15 |
| 13805 | 2 | C | 857.04 | 19.09 | 1.87 | 0.64 |
| 13827 | 1 | B | 589.22 | 16.44 | 1.49 | 3.07 |
| 13827 | 2 | C | 142.65 | 2.93 | 2.03 | 5.02 |
| 13827 | 3 | C | 138.71 | 2.58 | 2.24 | 5.15 |
| 13827 | 4 | C | 1928.74 | 66.93 | 1.20 | 0.27 |
| 13831 | 1 | B | 821.12 | 26.01 | 1.32 | 11.23 |
| 13871 | 1 | A | 87.78 | 2.38 | 1.54 | 11.87 |
| 13871 | 2 | A | 64.56 | 1.48 | 1.82 | 40.78 |
| 13871 | 3 | A | 102.52 | 3.56 | 1.20 | 65.92 |
| 13871 | 4 | A | 697.88 | 17.39 | 1.67 | 31.43 |
| 13871 | 5 | A | 119.27 | 3.30 | 1.51 | 105.05 |
| 13871 | 6 | A | 29.14 | 1.15 | 1.06 | 28.02 |
| 13915 | 1 | B | 333.95 | 5.27 | 2.64 | 30.28 |
| 13916 | 1 | A | 109.02 | 2.54 | 1.79 | 11.05 |
| 13916 | 2 | A | 1204.51 | 26.55 | 1.89 | 8.85 |
| 13916 | 3 | A | 93.90 | 1.35 | 2.91 | 60.37 |
| 13916 | 4 | A | 1277.47 | 27.71 | 1.92 | 28.02 |
| 13916 | 5 | A | 926.31 | 32.99 | 1.17 | 46.03 |
| 13916 | 6 | A | 201.72 | 3.94 | 2.14 | 17.73 |
| 13916 | 7 | A | 590.52 | 18.66 | 1.32 | 37.47 |
| 13916 | 8 | A | 433.50 | 11.57 | 1.56 | 82.75 |
| 13916 | 9 | A | 562.75 | 18.01 | 1.30 | 43.54 |
| 13922 | 1 | B | 1080.94 | 34.39 | 1.31 | 5.03 |
| 13922 | 2 | C | 143.13 | 4.60 | 1.30 | 4.68 |
| 13939 | 1 | B | 972.76 | 29.15 | 1.39 | 10.02 |
| 13939 | 2 | C | 208.84 | 4.46 | 1.95 | 7.62 |
| 13949 | 1 | B | 289.04 | 6.35 | 1.90 | 62.50 |
| 13949 | 2 | C | 184.99 | 4.28 | 1.80 | 12.37 |
| 13949 | 3 | B | 1131.60 | 50.53 | 0.93 | 35.52 |
| 13949 | 4 | A | 718.17 | 26.18 | 1.14 | 35.30 |
| 13949 | 5 | A | 624.55 | 27.33 | 0.95 | 35.50 |
| 13949 | 6 | A | 264.95 | 8.36 | 1.32 | 21.55 |
| 13949 | 7 | A | 288.46 | 15.33 | 0.78 | NA |
| 13969 | 1 | A | 94.51 | 1.47 | 2.68 | 140.53 |
| 13969 | 2 | A | 820.58 | 24.68 | 1.39 | 71.83 |
| 13969 | 3 | A | 1838.66 | 53.10 | 1.44 | 75.05 |
| 13978 | 1 | A | 224.62 | 3.05 | 3.07 | 24.90 |
| 13978 | 2 | B | 289.73 | 4.81 | 2.51 | 5.67 |
| 13978 | 3 | C | 1234.54 | 29.70 | 1.73 | 51.92 |
| 13978 | 4 | C | 202.86 | 3.95 | 2.14 | 5.27 |
| 13978 | 5 | C | 1260.77 | 26.68 | 1.97 | 57.10 |
| 13978 | 6 | C | 460.73 | 8.45 | 2.27 | 39.47 |
| 13978 | 7 | C | 828.51 | 15.99 | 2.16 | 61.63 |
| 13978 | 8 | C | 977.84 | 15.11 | 2.70 | 17.15 |
| 13978 | 9 | C | 799.40 | 10.33 | 3.22 | 8.54 |
| 13985 | 1 | B | 411.10 | 6.55 | 2.62 | 16.75 |
| 13985 | 2 | C | 548.90 | 12.95 | 1.77 | 12.18 |
| 13985 | 3 | C | 91.05 | 3.14 | 1.21 | 26.72 |
| 13985 | 4 | C | 47.04 | 1.94 | 1.01 | 7.07 |
| 13985 | 5 | C | 40.85 | 1.64 | 1.04 | 3.33 |
| 13985 | 6 | C | 21.05 | 1.47 | 0.60 | 41.07 |
| 13985 | 7 | C | 113.98 | 3.82 | 1.24 | 4.72 |
| 13985 | 8 | C | 99.81 | 2.76 | 1.51 | 25.87 |
| 13985 | 9 | C | 381.65 | 13.08 | 1.22 | 20.45 |
| 13985 | 10 | C | 2234.59 | 58.65 | 1.59 | 2.79 |
| 13988 | 1 | A | 1179.89 | 27.88 | 1.76 | NA |
| 14587 | 1 | B | 648.00 | 17.78 | 1.52 | 41.98 |
| 14587 | 2 | B | 1188.83 | 22.88 | 2.16 | 79.65 |
| 14587 | 3 | A | 755.52 | 22.35 | 1.41 | 71.55 |
| 14587 | 4 | A | 481.50 | 10.98 | 1.83 | 39.30 |
| 14587 | 5 | A | 407.79 | 9.65 | 1.76 | 33.80 |
| 14587 | 6 | A | 418.13 | 9.22 | 1.89 | 39.42 |
| 14587 | 7 | A | 140.61 | 5.25 | 1.12 | 30.92 |
| 14587 | 8 | A | 50.52 | 1.93 | 1.09 | 14.08 |
| 14587 | 9 | A | 429.20 | 10.32 | 1.73 | 27.50 |
| 14587 | 10 | A | 375.96 | 11.92 | 1.31 | 59.97 |
| 14587 | 11 | A | 375.19 | 9.98 | 1.57 | 90.76 |
